# Progressive encephalopathy associated with novel compound heterozygous NAXE mutations in a Chinese patient: case report and literature review

**DOI:** 10.3389/fped.2026.1766864

**Published:** 2026-02-09

**Authors:** Yanjie Zhu, Peifeng He, Rong Luo, Xiaolu Chen

**Affiliations:** 1 Department of Pediatrics, West China Second University Hospital, Sichuan University, Chengdu, China; 2 Key Laboratory of Birth Defects and Related Diseases of Women and Children, Sichuan University, Ministry of Education, Chengdu, China

**Keywords:** case report, NAD(P)HX epimerase gene, NAXE gene, neurometabolic disorder, progressive encephalopathy

## Abstract

**Background:**

NAD(P)HX epimerase (NAXE) deficiency is a rare, often fatal, autosomal recessive neurometabolic disorder of early childhood, characterized by acute neurological regression triggered by febrile illness. Here, we report a case with compound heterozygous NAXE mutations (c.733A > C and c.389A > C) associated with a milder phenotype, thereby expanding the known disease spectrum.

**Case report:**

A previously healthy 19-month-old girl presented with acute neurological regression after a high-grade fever, losing motor skills and exhibiting lethargy. Initial investigations showed leukocytosis, elevated C-reactive protein, and MRI findings of sulcal/cisternal widening and spinal cord signal changes. Given the unexplained encephalopathy, whole-exome sequencing was performed, which identified compound heterozygous NAXE mutations, confirming the diagnosis. Management included intravenous immunoglobulin, corticosteroids, and NAD + precursors. Neurological improvement was observed during the hospital course, and near-complete motor recovery was achieved by the 11-month follow-up.

**Conclusion:**

This case underscores the need to consider NAXE-related encephalopathy in children with fever-induced acute neurological decline. The discovery of a novel compound heterozygous variant combination [c.389A > C [p.His130Pro] and c.733A > C [p.Lys245Gln]] defines a milder phenotypic spectrum and mandates early genetic testing for timely diagnosis and prognostic insight. Importantly, given the single-case nature of this observation, conclusion regarding treatment efficacy remains hypothesis-generating and require validation in additional cases.

## Introduction

Mutations in the NAD(P)HX epimerase (NAXE) gene are responsible for a rare autosomal recessive neurometabolic disorder, previously known as progressive encephalopathy with brain edema and/or leukoencephalopathy-1 (PEBEL1; OMIM #617186) (1, 2). This life-threatening condition typically presents during infancy with acute neurological decline precipitated by febrile illness, and is characterized by symptoms such as ataxia, psychomotor regression, and respiratory failure, often leading to early death within the first few years of life (3, 4). The NAXE gene encodes a key enzyme within the NAD(P)HX repair system, which is essential for preventing the toxic buildup of hydrated NAD(P)H derivatives and for maintaining cellular redox homeostasis (2). Since its initial identification in 2016 (3), fewer than 40 cases have been reported worldwide, with the NAXE c.733A > C (p.Lys245Gln) variant being relatively common among them. The disorder exhibits a heterogeneous clinical spectrum, featuring early-onset ataxia, neurodevelopmental regression, seizures, respiratory failure, and high mortality, often precipitated by febrile illnesses or other metabolic stressors (1, 3). Available evidence indicates that NAXE-related disease demonstrates considerable phenotypic variability, ranging from rapidly progressive and fatal infantile encephalopathy to chronic, slowly progressive forms that may extend into adulthood (4–7). Nevertheless, due to the limited number of documented cases, genotype-phenotype correlations, long-term outcomes, and optimal treatment strategies remain poorly understood.

Here, we report a detailed case of a 19-month-old Chinese female with NAXE deficiency, compound heterozygous for c.733A > C and c.389A > C, who developed developmental regression, ataxia, and hypotonia after a febrile illness. Her clinical management included immunomodulatory and mitochondrial cofactor therapy, and on follow-up, she exhibited near-complete motor recovery. By comparing our patient with nine previously reported cases carrying the NAXE c.733A > C variant, we highlight the expanding clinical spectrum of this disorder and underscore the importance of early diagnosis and intervention to improve outcomes in affected individuals. This case contributes to the growing understanding of NAXE-related disorders and underscores the importance of including this condition in the differential diagnosis of children presenting with unexplained ataxia or acute neurological decline, particularly in the setting of febrile episodes.

## Case presentation

The chronological sequence of key clinical events, diagnostic considerations, and interventions is summarized in Table 1. A previously healthy 19-month-old girl was admitted with a 10-day history of intermittent high-spiking fever (peak 39.8 °C), accompanied by chills and rigors. During the convalescent phase of the febrile episode, she developed progressive neurological decline over one week, marked by profound lethargy and significant motor regression. She lost previously acquired milestones, including the ability to walk independently, sit without support, and maintain head control. Parental reports also described hypophonia, resting tremors, and perioral erythema. No seizures were observed. Initial laboratory studies at a referring hospital revealed leukocytosis (white blood cell count 28.12 × 10⁹/L, 79.6% neutrophils), significantly elevated inflammatory markers (C-reactive protein 109.86 mg/L), and sterile pyuria with numerous leukocytes and erythrocytes on urinalysis. Despite a two-day course of intravenous piperacillin-tazobactam, the fever persisted. The antibiotic was switched to cefoperazone-sulbactam, after which her temperature normalized.

**Table 1:** Timeline of diagnostic evaluation and management in a case of NAXE deficiency.

Time point	Clinical & diagnostic milestones	Therapeutic interventions	Key results
HD 1	Admission. Acute neuro-regression post-fever with meningism. Leading differential: bacterial meningitis.	Initiated: IV meropenem, HFNC, IV mannitol, IVIG.	Initial labs (metabolic panel, CK) normal. ABG: resp. acidosis with hyperlactatemia (2.32 mmol/L).
HD 2	Normal CSF rules out bacterial meningitis. Shift focus to viral encephalitis.	Added: IV acyclovir, B vitamins.	CSF normal. Nasopharyngeal PCR: adenovirus positive.
HD 3–4	Minimal improvement. Immune-mediated etiology considered.	Ongoing empiric therapy. Workup expanded.	Video-EEG: background slowing.
∼HD 5	Trial of immunomodulation initiated due to poor response.	Added: IV methylprednisolone. Sent: Autoantibody panels (CSF/serum), MRI brain/spine, NCS.	—
HD 7	Definite improvement (weaned from HFNC, louder voice, tremor resolved) but unable to sit independently. Pivotal results return.	Ongoing therapy.	Autoantibody panels negative. CSF PCRs negative. MRI: sulcal/cisternal widening & spinal cord T2 hyperintensities (atypical). NCS: reduced amplitudes.
HD 8	Infection & primary neuroinflammation excluded. Focus shifts to inborn error of metabolism.	D/C: Meropenem, acyclovir, methylprednisolone. Continued: B vitamins, methylcobalamin, physiotherapy. Sent: WES.	—
HD 9–21	Steady neurological recovery.	Supportive therapy.	—
Discharge	Sits independently, ambulates with support. Meningeal signs resolved.	Discharged on B vitamins, methylcobalamin.	—
Post-discharge	Molecular diagnosis confirmed.	Initiated (outpatient): Nicotinamide and Coenzyme Q10	WES: NAXE c.389A > C & c.733A > C (comp het).
11-month FU	Near-complete recovery: independent ambulation, age-appropriate speech.	Nicotinamide and Coenzyme Q10	—

HD, hospital day; HFNC, high-flow nasal cannula; ABG, arterial blood gas; CK, creatine kinase; CSF, cerebrospinal fluid; IV, intravenous; IVIG, intravenous immunoglobulin; MRI, magnetic resonance imaging; NCS, nerve conduction studies; PCR, polymerase chain reaction; resp. acidosis, respiratory acidosis; Video-EEG, video-electroencephalography; D/C, discontinued; WES, whole-exome sequencing; comp het, compound heterozygous; FU, follow-up.

The patient was born at full term via spontaneous vaginal delivery to non-consanguineous parents, with an uncomplicated perinatal course. She was the second child and had achieved all developmental milestones appropriately, including independent walking by 12 months and had acquired a vocabulary of 15–20 words by the time of presentation. Family history was significant for the unexplained death of one sibling at 16 months of age; no autopsy was performed, and a definitive diagnosis remained unknown.

Upon admission to our institution, physical examination revealed a drowsy but arousable toddler. Vital signs included tachycardia (heart rate 150 bpm) and tachypnea (respiratory rate 46/min), with a normal body temperature. Cutaneous inspection showed prominent perioral erythema with a well-demarcated border. Neurological examination demonstrated signs of meningeal irritation (neck stiffness, positive Kerning's sign), bilateral Babinski responses, and profound axial and appendicular hypotonia with a frog-leg posture; She was unable to lift her head or maintain antigravity positions.

Given the acute presentation with meningism (objective findings of neck stiffness and positive Kerning's sign) and a history of marked leukocytosis, our initial leading differential diagnosis on hospital day 1 was acute bacterial meningitis. We commenced empirical therapy with intravenous meropenem (40 mg/kg/dose every 8 h). Supportive measures, including oxygen therapy via high-flow nasal cannula (HFNC) and intravenous mannitol (2.5 mL/kg every 6 h) for suspected intracranial hypertension, were initiated concurrently. Considering the possibility of a post-infectious inflammatory state, a single course of intravenous immunoglobulin (2 g/kg total dose) was also administered. The initial presentation with high fever, sterile pyuria, and elevated inflammatory markers also prompted consideration of Kawasaki disease; however, echocardiography performed at this time was normal, effectively ruling out coronary artery involvement associated with this condition. Initial laboratory investigations, including a comprehensive metabolic panel (blood glucose, electrolytes, renal and liver function, ammonia), creatine kinase, and arterial blood gas analysis, were performed. The metabolic panel and creatine kinase were within normal limits. Arterial blood gas revealed respiratory acidosis with mild hyperlactatemia (2.32 mmol/L). Together, these findings argued against common acute metabolic encephalopathies such as hepatic, uremic, hypoglycemic, or severe hyperammonemic causes.

By hospital day 2, cerebrospinal fluid analysis results showed normal (no pleocytosis, normal protein and glucose, negative Gram stain), effectively excluding active bacterial meningitis. With bacterial infection ruled out, diagnostic consideration shifted to viral encephalitis as the next most likely etiology, leading to the addition of intravenous acyclovir (10 mg/kg/dose every 8 h). Empirical supplementation with B vitamins (B1 10 mg, B2 5 mg, B6 10 mg daily) was initiated as supportive therapy for encephalopathy of unknown etiology.

Over the next several days (approximately days 3–4), subtle and inconsistent improvements in alertness and vocalization were noted, but her overall neurological deficit remained profound. A video-EEG showed generalized background slowing. The lack of substantial improvement prompted suspicion for an immune-mediated process. To further evaluate this possibility, a series of diagnostic investigations were undertaken, including brain and spinal magnetic resonance imaging (MRI), nerve conduction studies, and comprehensive autoantibody panels in both cerebrospinal fluid (CSF) and serum, targeting antigens associated with autoimmune encephalitis, demyelinating disorders, paraneoplastic syndromes, and oligoclonal band analysis. Concurrently, and in consideration of the suspected pathophysiology, immunomodulatory therapy was initiated with intravenous methylprednisolone at a dose of 1 mg/kg twice daily.

**Figure 1:**
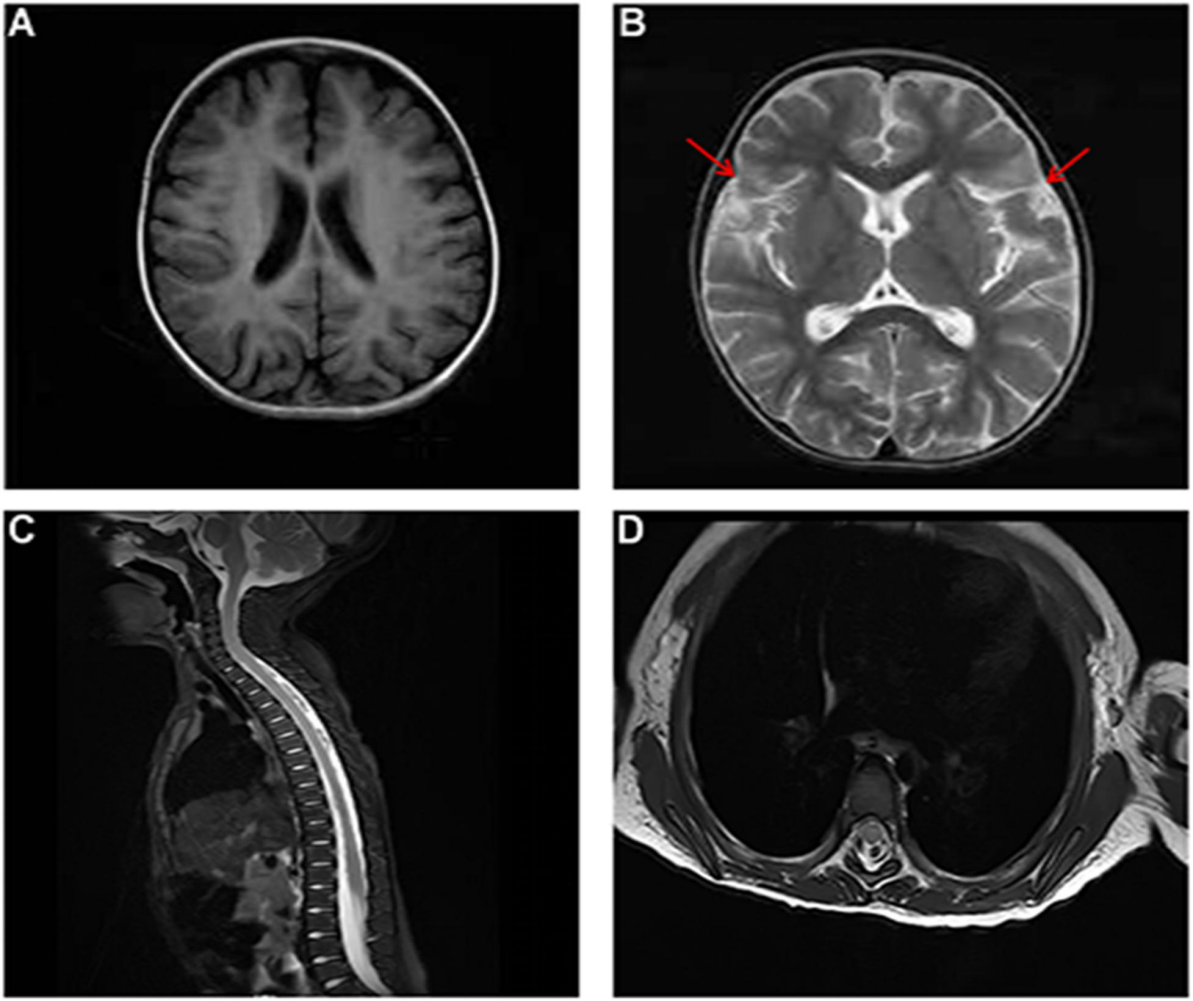
Brain and spinal cord magnetic resonance imaging (MRI) findings. **(A,B)** Axial brain MRI sequences [**(A)** T1-weighted; **(B)** T2-weighted] demonstrate sulcal and cisternal widening (red arrows). **(C,D)** Sagittal and axial spinal MRI sequences reveal a heterogeneous signal intensity and subtle T2 hyperintensity within the cervicothoracic spinal cord.

By hospital day 7, a more definite and sustained clinical improvement was observed. Notable positive changes included successful weaning from ventilatory support, improved vocal strength, resolution of resting tremors, and enhanced alertness; however, the patient remained significantly disabled and was still unable to sit independently. Concurrently, results from pending investigations became available. Comprehensive autoantibody panels and cerebrospinal fluid (CSF) pathogen PCR assays were negative. Brain and spinal MRI revealed sulcal and cisternal widening along with cervicothoracic spinal cord T2 hyperintensities (Figure 1), a pattern not consistent with typical acute disseminated encephalomyelitis (ADEM), autoimmune limbic encephalitis, or transverse myelitis. Nerve conduction studies demonstrated asymmetrically reduced amplitudes. Collectively, these findings ruled out active central nervous system (CNS) infection and a primary autoimmune or inflammatory demyelinating disorder as underlying etiologies. The observed clinical improvement was thus considered potentially attributable to supportive care, the natural course of the condition, intensive rehabilitation, or a combination of these factors. With common acute acquired etiologies excluded, the diagnostic focus shifted definitively toward an inborn error of metabolism, prompting initiation of whole-exome sequencing.

**Figure 2:**
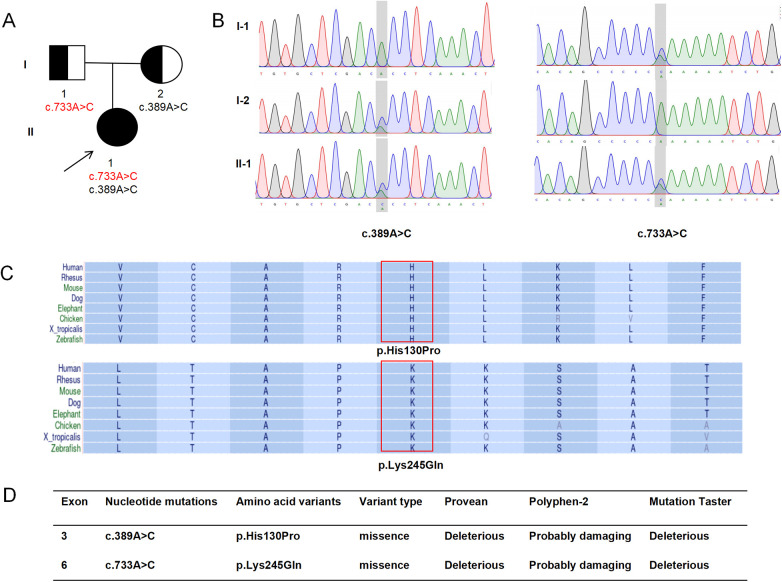
Genetic and in silico analysis of NAXE variants. **(A)** Pedigree of the family. The arrow indicates the proband. The segregation pattern confirms that the two NAXE variants were inherited separately from each parent Pedigree of the family showing segregation of the NAXE variants. **(B)** Sanger sequencing chromatograms validating the compound heterozygous NAXE variants (c.389A > C and c.733A > C) in the proband. **(C)** Cross-species conservation analysis of the amino acid residues affected by the NAXE variants (His130 and Lys245), demonstrating high evolutionary conservation. **(D)** Computational assessment of variant impact. Predictions from multiple in silico tools (PROVEAN, PolyPhen-2, MutationTaster) classified both variants as “deleterious” or “probably damaging”, supporting their potential pathogenicity (PP3 evidence).

Her neurological function demonstrated progressive improvement over the subsequent two weeks. At the time of discharge, meningeal signs had resolved. She had regained substantial motor strength, was able to sit independently, and could ambulate with assistance. Whole-exome sequencing, performed prior to discharge but with results available only afterward, revealed compound heterozygous pathogenic variants in the NAXE gene (NM_144772.3) [c.389A > C (p.His130Pro) and c.733A > C (p.Lys245Gln)], supporting the diagnosis of NAXE deficiency (Figure 2B). Targeted treatment with nicotinamide and coenzyme Q10 were initiated in the outpatient setting following molecular confirmation. At the 11-month follow-up, she exhibited near-complete neurological recovery. She had regained independent walking and could speak in short sentences. Fine motor skills were slightly delayed but showed continued improvement. No focal neurological deficits, ataxia, or pyramidal signs were observed on examination. Given her favorable clinical status and absence of new symptoms, repeat neuroimaging (MRI) or neurophysiological studies (EEG/NCS) were not performed during this follow-up, consistent with shared decision-making with her family.

**Figure 3:**
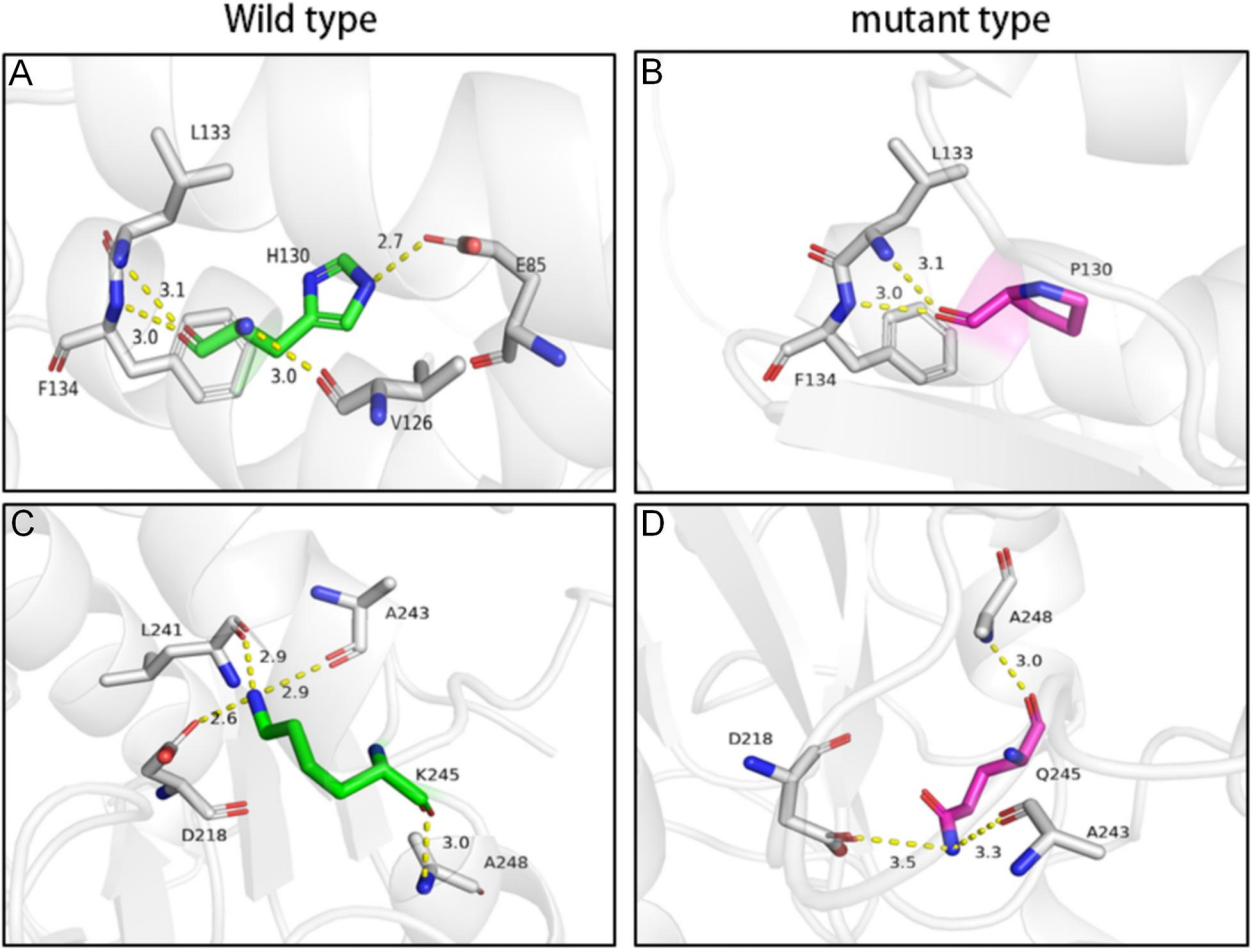
Structural modeling of wild-type and mutant NAXE protein. **(A,C)** Wild-type (WT) structures showing the native conformation and key stabilizing interactions (yellow dashed lines) around residues His130 **(A)** and Lys245 **(C)**, respectively. **(B,D)** Mutant structures modeling the p.His130Pro **(B)** and p.Lys245Gln **(D)** variants. The substitutions are predicted to disrupt local interactions and compromise structural stability.

Evolutionary conservation analysis demonstrated that both His130 and Lys245 are highly conserved residues across species (Figure 2C). Computational pathogenicity prediction tools, including PROVEAN, PolyPhen-2, and MutationTaster, consistently classified both variants as “deleterious” or “probably damaging” (Figure 2D), providing PP3 evidence (multiple computational predictions support a deleterious effect). According to the American College of Medical Genetics and Genomics (ACMG) guidelines, both variants were classified as Variants of Uncertain Significance (VUS). The novel c.389A > C variant was classified based on evidence codes PM2_Supporting (absent or at very low frequency in population databases; specifically, this variant is not present in gnomAD v3.1.2 with a minor allele frequency <0.0001) and PP3 (multiple computational predictions support a deleterious effect; PROVEAN score −5.82, PolyPhen-2 score 0.998, and MutationTaster probability 0.99 all classify this variant as deleterious/probably damaging). The c.733A > C variant, previously reported in trans configuration with other pathogenic variants in NAXE deficiency patients, was classified based on PM3_Strong (identified in trans with another variant in an autosomal recessive disorder) and PP3 evidence. Three-dimensional structural modeling performed with PyMOL indicated that these missense mutations are likely to disrupt native hydrogen-bonding networks with surrounding residues, thereby compromising NAXE protein stability (Figure 3). In the context of the patient's classic phenotype for NAXE deficiency, the finding of these compound heterozygous variants (in trans) was considered diagnostic, despite their individual VUS classifications. We acknowledge that functional validation of these specific variants is not yet available, which represents a potential limitation of this study.

## Literature review

We conducted a targeted literature search of the PubMed, Wanfang, and CNKI databases using the keywords “NAXE”, “APOAIBP”, “NAD(P)HX epimerase”, and “AIBP” to identify all previously reported cases harboring the NAXE c.733A > C variant. Publications were included if they provided sufficient detail for phenotypic and genotypic characterization. As of September 2025, a total of 10 cases (including the present one) carrying this variant have been reported worldwide. Comprehensive analysis of these genetically confirmed cases reveals distinct clinical patterns associated with this rare neurometabolic disorder. The genetic characteristics and clinical manifestations of patients with NAXE c.733A > C mutations are summarized in Table 2.

**Table 2:** Clinical features of our case and 9 previously reported cases harboring the c.733A > C variant.

No.	Ref.	Gender	Nationality	NAXE mutation	Age at onset	Inducement	Clinical characteristics	MRI features	Lactate level （CSF/blood）	EEG	Outcome	Treatments
1	This study	Female	Chinese	c.733A > C c.389A > C	1 yr 7 mo	Fever	Developmental regression, drowsiness, ataxia, tremor, hypotonia, dysarthria	Prominent sulcal/cisternal widening, signal changes within the spinal cord	Blood lactate: 2.32 mmol/L	Background slowing	Stable	Antibiotic and antiviral therapy, IVIG, corticosteroids, vitamin B complex (B1, B2, B3, B6, B12), coenzyme Q10
2	(5)	Male	South African	c.733A > C	1 yr 3 mo	Fever	Chronic ataxia, nystagmus, strabismus, ptosis, dysarthria, mild spasticity of lower limbs, developmental regression, neuropsychiatric symptoms	Cerebellar and spinal cord atrophy, reduced NAA peak on MRS	CSF lactate: 3.0 mmol/L	NR	Stable with disability	Vitamins B1, B2, B3, carnitine, coenzyme Q10
3	(21)	Female	Chinese	c.733 A > C	1 yr 8 mo	Fever	Psychomotor regression, hypotonia, hand tremors, consciousness disturbance, respiratory failure, ataxia, seizures, strabismus, dysphagia, dysarthria	Symmetric hyperintensity in middle cerebellar peduncles; later cerebellar atrophy	Normal (CSF)	Background slowing	Stable with disability	IVIG, steroids, coenzyme Q10, vitamin B complex, carnitine, anti-epileptics
4	(13)	Female	Chinese	c.733A > C c.370G > T	2 yr 10 mo	Fever, rabies vaccination	Respiratory failure, psychomotor regression, coma, gait instability, hypotonia, ptosis, limb weakness, dysphagia, nystagmus	Early stage: Normal brain MRI; T7–T10 spinal cord T2 hyperintensity Later stage: Diffuse cerebellar swelling, hydrocephalus, brainstem/cervical cord lesions	Normal (blood)	Background slowing, bilateral temporal spikes/polyspikes	Died	Antiviral, IVIG, steroids, plasma exchange, vitamin B3, coenzyme Q10, levocarnitine,
5	(4)	Male	Saudi Arabian	c.733A > C	NR	NR	Respiratory failure, motor delay, dystonia, septicemia	NR	NR	NR	NR	NR
6	(18)	Female	Korean	c.733A > C c.368A > T	2 yr 8 mo	Fever	Psychomotor regression, respiratory failure, hypotonia,seizures, ataxia, tremors, nystagmus, dysphagia, dysarthria, nephrotic syndrome	Symmetric T2-hyperintense lesions: cerebellar cortex, brainstem, cerebral peduncles, medial temporal areas; later severe cerebellum and brainstem atrophy	NR	NR	Stable with disability	NR
7	(22)	Female	Chinese	c.733A > C	2 yr	Fever	Psychomotor regression, hypotonia, limb weakness, ataxia, tremors, strabismus, dysarthria, respiratory failure, episodic deterioration, nystagmus, strabismus, dysphagiarash	Early stage: Normal Later stage: Symmetric abnormalities in cerebellum/brainstem, white matter lesions, brain edema	Normal (blood and CSF)	Slow-wave activity (6–8 Hz), asymmetric amplitudes, intermittent spikes/sharp waves	Died	IVIG, steroids, Coenzyme Q10, L-carnitine
8	(19)	Female	Chinese	c.733A > C	2 yr 8 mo	Fever	Psychomotor regression, seizures, tremors, hypotonia, ataxia, dysarthria, respiratory failure, coma	Early stage: Symmetric lesions in middle cerebellar peduncles & hemispheres; spinal cord swelling (C-T segments). Later stage: Cerebellar and spinal lesions resolved; leptomeningeal enhancement present.	Normal (blood)	Diffuse high-amplitude slow waves	Stable with disability	IVIG, steroids, Coenzyme Q10, L-carnitine, idebenone, vitamin B2, B3 and antiepileptics
9	(19)	Male	Chinese	c.733A > C	1 yr 5 mo	Fever	Psychomotor regression, hypotonia, seizures, tremors, respiratory failure, coma, ataxia, central diabetes insipidus	Initially normal → non-specific cerebral white matter lesions → cervical/upper thoracic spinal cord swelling → widespread cerebral lesions (basal ganglia, white matter) and atrophy in final stage.	Blood lactate: 2.28 mmol/L	Diffuse high-voltage slow waves; epileptiform discharges	Died	Mechanical ventilation, steroids, IVIG, antiepileptics, antiviral and vitamins B1, B6, methylcobalamin
10	(17)	Female	Chinese	c.733A > C c.304_c.305insA	1 yr 8 mo	Fever	Respiratory failure, psychomotor regression, coma, hypotonia,ataxia, dysarthria	Spinal cord T2 hyperintensity, cerebellar bar-like hyperintensities	Normal (blood)	NR	Died	IVIG, steroids, plasma exchange, CVVH, vitamin B3

Ref., Reference; yr, year; mo, month; MRI, magnetic resonance imaging; MRS, magnetic resonance spectroscopy; NAA, N-acetylaspartate; CSF, cerebrospinal fluid; EEG, electroencephalogram; IVIG, intravenous immunoglobulin; CVVH, continuous Veno-venous hemofiltration; NR, not reported.

The clinical features of our patient and the nine previously reported cases are as follows. Age of onset was consistently early, occurring before 3 years of age in all patients with available data (90%, 9/10), with the majority of episodes (90%, 9/10) triggered by febrile illness. The most common clinical manifestations included psychomotor regression (100%, 10/10), dystonia (100%, 10/10), ataxia (100%, 10/10), respiratory failure (80%, 8/10), dysarthria (70%, 7/10), tremors (60%, 6/10), seizures (50%, 5/10), dysphagia (40%, 4/10), nystagmus (40%, 4/10), strabismus (30%, 3/10), and ptosis (20%, 2/10). A skin rash was reported in one case (10%, 1/10).

Brain or spinal MRI findings were reported for 9 of the 10 patients (with one case lacking detailed data). Among these 9 patients, the observed abnormalities included: cerebellar or brainstem lesions (77.8%, 7/9), spinal cord involvement (77.8%, 7/9), brain atrophy (55.6%, 5/9), white matter signal changes (22.2%, 2/9), and brain edema (22.2%, 2/9). Elevated lactate levels were identified, with two cases involving the blood and one involving the CSF. Among the six patients with available EEG results, half exhibited background slowing and half demonstrated epileptiform discharges.

The outcome was fatal in 4 cases, while 5 patients were reported to be stable, though often with severe disabilities; the outcome was unknown in one case. Treatment strategies commonly included immunomodulatory agents [e.g., intravenous immunoglobulin (IVIG), steroids], vitamin supplements (particularly B vitamins), and metabolic therapies such as coenzyme Q10 and carnitine. Notably, Cases 8, 9, and 10 presented with a clinical and neuroimaging phenotype consistent with Leigh syndrome, characterized by symmetric bilateral lesions in the brainstem, basal ganglia, and/or cerebellar regions, along with progressive neurological deterioration.

Collectively, the ten reported cases carrying the NAXE c.733A > C variant delineate a recognizable clinical phenotype, characterized by early-onset encephalopathy triggered by febrile illness (Table 2). Nevertheless, NAXE deficiency as a whole exhibits substantial phenotypic and genetic heterogeneity. From a genetic perspective, individuals outside the c.733A > C group demonstrate greater diversity in mutation type, featuring a broader spectrum of loss-of-function variants that include frameshift, splice-site, and nonsense mutations distributed throughout the gene (1, 4, 6, 7). Phenotypically, although overlapping features exist, certain NAXE genotypes are linked to broader systemic involvement. Notably, some patients develop cardiomyopathy and severe dermatological manifestations resembling pellagra (2, 8), the latter being a hallmark feature in NAXD deficiency where it is often more severe and prevalent (9–12).

Furthermore, specific variant combinations (e.g., p.Gly253Ser and c.665-1G > A) define a distinct later-onset form, presenting in adolescence or adulthood with psychiatric symptoms, chronic ataxia (4). Beyond the paradigmatic febrile trigger, isolated reports have suggested other potential precipitants in susceptible individuals, including metabolic stressors (2), substance exposures (e.g., alcohol, tetrahydrocannabinol) (4), and a temporal association with vaccination (13). These non-febrile associations remain anecdotal and require systematic validation. The striking recovery in our patient, compound heterozygous for c.389A > C (p.His130Pro) and c.733A > C, underscores this phenotypic diversity and highlights the potential for milder, treatable trajectories within the NAXE spectrum.

## Discussion

PEBEL1 (OMIM #617186) is a rare autosomal recessive neurometabolic disorder caused by pathogenic variants in the NAXE gene (formerly known as APOA1BP) located on chromosome 1q22 (14). The encoded enzyme plays an essential role in maintaining NAD(P)H homeostasis within neural tissues by catalyzing the interconversion between S- and R-NAD(P)HX stereoisomers (15). A hallmark clinical feature of this condition, progressive encephalopathy with brain edema and/or leukodystrophy (PEBEL), is acute neurological deterioration triggered by febrile illness, resulting from impaired enzymatic function under physiological stress (16). During fever or acidosis, the spontaneous hydration of NADH/NADPH is accelerated, overwhelming the compromised NAD(P)HX repair system and leading to the accumulation of neurotoxic metabolites (2). This well-defined pathophysiological cascade explains the characteristic vulnerability to febrile episodes and the rapid neurological decline observed in typical cases. In contrast to this often-severe presentation, the current case illustrates a previously under-recognized, more favorable outcome within the clinical spectrum of NAXE deficiency: a patient who achieved near-complete functional recovery after a fever-induced acute encephalopathy.

The diagnostic process in this case highlights the critical importance of considering inborn errors of metabolism in children presenting with acute acquired encephalopathy. Following the systematic exclusion of common acquired etiologies, including infectious, autoimmune, and demyelinating conditions, whole-exome sequencing emerged as the pivotal step toward achieving a definitive diagnosis. In infants and young children who exhibit acute, unexplained neurological regression following febrile episodes, NAXE deficiency should be included in the differential diagnosis, even when clinical manifestations resemble those of infection or inflammation. Early genetic testing is essential, as it not only terminates the prolonged diagnostic journey but also informs subsequent therapeutic strategies and prognostic counseling.

The genotype-phenotype correlation in this case warrants further discussion. The patient is compound heterozygous for the c.733A > C and c.389A > C variants. The c.733A > C variant is common among reported cases, but its clinical outcome largely depends on the type of its paired allele. When combined with severe loss-of-function variants, it often leads to rapid progression and death (13, 17). However, in this case, its combination with another missense variant, c.389A > C, is associated with significant neurological recovery. We hypothesize that this specific pair of missense variants may retain partial enzymatic activity, thereby enabling metabolic compensation, especially with precursor supplementation. This significantly expands our understanding of the phenotypic spectrum of NAXE deficiency, which ranges from fatal infantile Leigh-like encephalopathy to chronic ataxia with onset in adulthood (5, 18, 19), and now to this treatable benign course as reported here.

The issue of cutaneous manifestations requires clarification based on the latest evidence. The previous view held that severe skin involvement (e.g., erythematous necrotic plaques in flexural areas) was characteristic of NAXD deficiency. However, a recent systematic review indicates that characteristic skin lesions are observed in up to one-third of patients with NAXE deficiency as well (20). The “perioral erythema” observed in our patient, although atypical, suggests that NAXE deficiency may also involve a broader multisystemic spectrum, which neurologists should note during evaluation.

The diagnostic process in this case highlights the critical importance of considering inborn errors of metabolism in children presenting with acute acquired encephalopathy. Neuroimaging findings in NAXE deficiency show dynamic evolution and provide valuable diagnostic clues. Although initial MRI may be unremarkable, follow-up studies often demonstrate symmetrical cerebellar edema, brainstem tegmental abnormalities, and T2-weighted hyperintensities along the spinal cord, which may progress to irreversible atrophy. Notably, the absence of elevated CSF lactate in most cases helps differentiate NAXE deficiency from classical mitochondrial disorders, underscoring a distinct metabolic pathogenesis. The discrepancy between normal lactate levels and profound neurological dysfunction suggests alternative mechanisms, such as energy failure or oxidative stress, may be involved (13, 21). Following the systematic exclusion of common acquired etiologies, including infectious, autoimmune, and demyelinating conditions, whole-exome sequencing emerged as the pivotal step toward achieving a definitive diagnosis. In infants and young children who exhibit acute, unexplained neurological regression following febrile episodes, NAXE deficiency should be included in the differential diagnosis, even when clinical manifestations resemble those of infection or inflammation. Early genetic testing is essential, as it not only terminates the prolonged diagnostic journey but also informs subsequent therapeutic strategies and prognostic counseling.

Regarding treatment, improvement was observed during a period when the patient received a comprehensive regimen involving immunomodulation, NAD + precursor supplementation, and intensive rehabilitation. However, attribution of effect must be cautious. Given the potentially fluctuating natural history of the disease, the possibility of spontaneous recovery, and the single-case nature of this observation without controlled comparison, we cannot establish causality or determine which specific intervention, if any, was predominant. The observed clinical improvement was thus considerable potentially attributable to supportive care, the natural course of the condition, intensive rehabilitation, or a combination of these factors. The literature shows inconsistent responses to immunomodulatory therapy, suggesting neuroinflammation may be a variable secondary component. In contrast, nicotinamide supplementation is theoretically targeted at the core metabolic defect and has been temporally associated with clinical improvement in several reports (2, 6). Therefore, this case suggests that for such rare metabolic encephalopathies, a pragmatic “combined strategy” addressing both potential secondary inflammation and the core metabolic defect is reasonable, though the efficacy of individual components remains unproven. To move beyond anecdotal evidence and established definitive treatment protocols, future research must utilize novel tools such as the newly established NAXE-knockout iPSC model (16) to screen and validate treatment strategies *in vitro*, ultimately clarifying the efficacy of individual interventions through controlled studies.

In summary, this case expands the clinical and genetic spectrum of NAXE deficiency by reporting a novel compound heterozygous variant combination associated with a favorable prognosis. It alerts neurologists that genetic testing should be considered in cases of fever-induced acute neurological regression, even when recovery is good. A definitive diagnosis helps guide patient management (e.g., considering long-term niacin/nicotinamide supplementation, aggressive fever control) and provides accurate prognostic counseling. Further research is needed to elucidate genotype-phenotype correlations and establish evidence-based treatment protocols.

## Conclusion

This case report expands the clinical and genetic spectrum of NAXE deficiency. The identification of a novel compound heterozygous variant combination [c.389A > C [p.His130Pro] and c.733A > C [p.Lys245Gln]] underscores the disorder's phenotypic heterogeneity, which can include milder presentations with significant recovery potential. Our findings highlight the critical importance of early genetic testing in children with fever-induced acute neurological decline of unknown origin. A timely diagnosis is essential to guide appropriate management and provide accurate prognostic counseling. Further research is warranted to establish definitive genotype-phenotype correlations and evidence-based therapies. Importantly, given the single-case nature of this observation, conclusions regarding treatment efficacy remain hypothesis-generating and require validation in additional cases.

## Data Availability

The original contributions presented in the study are included in the article/Supplementary Material, further inquiries can be directed to the corresponding author.
